# Prevalence of Presenting Conditions in Grey Seal Pups (*Halichoerus grypus*) Admitted for Rehabilitation

**DOI:** 10.3390/vetsci2010001

**Published:** 2015-01-05

**Authors:** Marc A. C. Silpa, Susan M. Thornton, Tamara Cooper, Joanna Hedley

**Affiliations:** 1 Royal (Dick) School of Veterinary Studies, University of Edinburgh, Easter Bush Campus, Roslin, EH25 9RG, UK; 2 International Zoo Veterinary Group, Station House, Parkwood Street, Keighley, West Yorkshire, BD21 4NQ, UK; E-Mail: S.Thornton@izvg.co.uk; 3 Cornish Seal Sanctuary, Gweek, Nr. Helston, Cornwall, TR12 6UG, UK; E-Mail: Tamara.Cooper@merlinentertainments.biz; 4 Beaumont Sainsbury Animal Hospital, Royal Veterinary College, Royal College Street, London, NW1 0TU, UK; E-Mail: jhedley@rvc.ac.uk

**Keywords:** wildlife, pinniped, rehabilitation, epidemiology

## Abstract

A retrospective survey was performed on the presenting conditions of 205 live grey seal pups (*Halichoerus grypus*) admitted to the Cornish Seal Sanctuary in Gweek, United Kingdom between May 2005 and March 2011. The purpose of the survey was to examine the prevalence of various presenting signs at the sanctuary. The presenting signs were classified into nine non-mutually exclusive categories: ocular disorders, nasal disorders, oral disorders, respiratory disorders, orthopaedic disorders, puncture wounds, abrasions, netting injuries, and onychia. The sex ratio of seal pups in this study was 1.35 males per female. Of the 205 examined for rehabilitation, 22 (10.73%) did not survive to release. 68.78% of grey seal pups presented with puncture wounds, 47.80% with respiratory disorders, 46.34% with ocular disorders, 42.63% malnourished, 36.59% with abrasions, 25.37% with oral disorders, 23.90% with nasal disorders, 11.71% with orthopaedic disorders, 9.27% with onychia, and 3.41% presented with netting injuries. 52% were normothermic, 42% were hyperthermic, and 5% were hypothermic. Associations between gender, outcome of rehabilitation, hospitalisation time and presenting disorders were examined. In addition, admissions rates were found to display seasonality. The results of this study will aid in future preparation of grey seal rehabilitation facilities.

## 1. Introduction

Grey seals (*Halichoerus grypus*) are common among the shores of United Kingdom (UK) and frequently rehabilitated. The most recent estimate for the total grey seal UK population is between 90,600 and 142,900 [1].

Barnett *et al.* [2] first reported presenting conditions of grey seal pups in UK rehabilitation centres, using data dating from 1992 until 1998. Since that time, a major European outbreak of phocine distemper virus has affected the UK seal population [3,4]. Additionally, climate change and other anthropogenic activities have been implicated in changes to disease patterns of marine mammals [5,6] and other wildlife [7]. With continual changes in disease patterns, it is important for the UK to maintain a well-stocked and informed stranding network for seals. To achieve this goal, rehabilitation centres must be kept aware of expected diseases and conditions. The main purpose of this study was to examine the current prevalence of presenting signs in grey seal pups.

## 2. Methods

### 2.1. Seals

Records were examined from all juvenile grey seals which were admitted to the Cornish Seal Sanctuary in Gweek, UK from May 2005 until March 2011. Seals were rescued from along the south-west English coast by trained personnel. Some, but not all, seals were given oral rehydration fluids during rescue depending on the assessment and capability of the rescuer. Following a 30 min resting period post-transport, each seal was given a systematic physical examination upon admission to the sanctuary. The examination was performed by a trained animal handler or veterinary surgeon, and the findings were documented. Body condition score (BCS) was subjectively ranked as good, fair or poor.

### 2.2. Data

Admission records were entered into an Excel^®^ [8] database format. Age estimation was replaced with age classification based on lanugo coat pattern and umbilicus status as described by Barnett *et al.* [2]: neonate (lanugo coat present, umbilicus present), white-Coat (lanugo coat present, umbilicus not present), mid-moult (lanugo coat partially shed), and moulted (lanugo coat completely shed). Based on wild studies of grey seal pups, neonates are assumed to be younger than five days old, white-coats between five and 14 days old, mid-moult from 16 until 21 days, and pups are moulted from 11 days until 10 months old [9,10]. Temperature was classified as marked hypothermic, mildly hypothermic, normal, mildly hyperthermic, and marked hyperthermic based off reported normal values for grey seals [11]. The presenting signs were classified into nine non-mutually exclusive categories: ocular disorders, oral disorders, nasal disorders, respiratory disorders, orthopaedic disorders, puncture wounds, abrasions, netting injuries, and onychia. Trauma was separated into four categories based on type of wound and anatomical involvement: orthopaedic, puncture, abrasion and wounds caused by nets. Due to the inability to perform extensive radiographic investigation, any injuries that grossly included a bone or any obvious fractured bones were classified as an orthopaedic disorder. Superficial dermal wounds were classified as abrasions. Onychia included any inflammation around the nail. A seal was classified as malnourished if it had a poor BCS. Nasal disorders included nasal discharge. A respiratory disorder was indicated if there was marked effort in respiration or production of respiratory noise. Any lesion in the mouth was classified as an oral lesion.

There were two outcomes of rehabilitation. Either the seal was released or the seal did not survive to release. Any deaths that occurred after release were not considered due to the inability to secure accurate survival reports. Any humane euthanasia of seals at the centre was performed by injecting a lethal dose of pentobarbital intravenously. Hospitalisation time was calculated in days starting from day of admission until day of release or death.

To examine if hospitalisation admission displayed seasonality, a cosinor test was performed using R [12] as described by Barnett and Dobson [13]. For all other statistical calculations, Minitab^®^ version 16 [14] was used. Prevalence with 95% confidence intervals was calculated based on a binary examination of the classification disorders. To examine if age or gender were risk factors for the presenting conditions, univariable binary logistic regression analyses were performed. A similar analysis was also performed on outcome with presenting conditions and other variables. Variables with associations of *p* < 0.25 were placed into a multivariable binary logistic regression model, and the least significant variable excluded until a final statistically significant model was obtained.

## 3. Results

Between May 2005 and March 2011, a total of 205 grey seal pups were admitted for rehabilitation at the Cornish Seal Sanctuary (Table 1). BCS was reported in 190 pups. Only 22 pups did not survive until release. Of those 22, only one was euthanized. The seal pup was euthanized after 4.5 months due to worsening neurological signs and poor prognosis. Due to the low number neonates (*n* = 11), neonates and white-coat were combined for statistical analysis.

**Table 1:** Counts of grey seal pup age and gender.

	Neonate	White-Coat	Mid-Moult	Moulted	Total
Female	7	17	11	52	87
Male	4	11	19	84	118
Total	11	28	30	136	205

### 3.1. Prevalence

**Figure 1:**
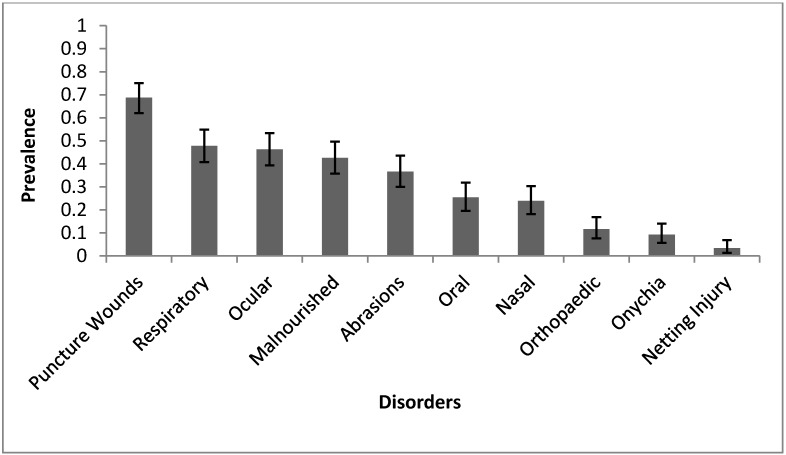
The prevalence and the 95% confidence interval (CI) of each presenting condition observed in rehabilitated grey seal pups. Total for all conditions = 205 pups except malnourishment. Only 190 pups had body condition score (BCS) reported.

The prevalence of each presenting disorder with 95% confidence intervals is shown on Figure 1. The most common presenting condition was trauma. 81.95% of seal pups had at least one form of trauma. 68.78% of seal pups had puncture wounds while 36.59% had abrasions. 11.71% had obvious orthopaedic disorders, and 3.41% has netting injuries. After trauma, respiratory disorders were the second most common presenting sign (47.80%). Ocular disorders (46.34%), malnourishment (42.63%), and oral lesions (25.37%) were also significantly present in this population. Onychia (9.27%) and netting injuries were the least common presenting conditions.

### 3.2. Gender

The significant univariable binary logistic regressions (Table 2) demonstrate that gender is associated with some of the presenting disorders. Females are significantly less likely to have oral disorders and abrasions. In addition, there is a trend of females being half as likely to present with respiratory disorders as males.

### 3.3. Age

Age was significantly associated with four presenting disorders (Table 2). Younger pups were less likely to have respiratory signs while white-coat pups were significantly less likely to present with nasal disorders, oral disorders and puncture wounds. Seven moulted pups present with netting injuries while no non-moulted pup (neonate, white-coat, and mid-moult) had these injuries. A fisher exact test showed a trend towards an association of age and netting injuries (*p* = 0.097).

### 3.4. Season

**Figure 2:**
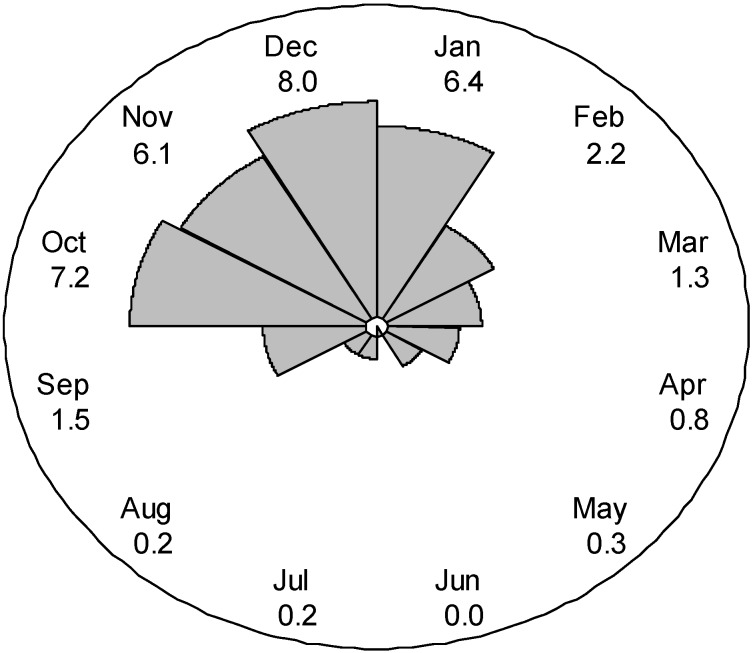
A rose diagram of monthly mean admission rates of grey seal pups from May 2005 through March 2011. Monthly means were adjusted for unequal number of days in each month.

Hospitalisation admissions occurred mainly between October and January (Figure 2). A cosinor test demonstrated that hospital admissions displayed seasonality (*p* < 0.001). The phase of the cosinor test was 11.7, indicating that the peak admissions was during late November. The lowest point of seal admissions occurred in late May.

**Table 2:** Univariable Binary Logistic Regressions of each presenting disorder observed in rehabilitated grey seal pups.

Disorder	Factor	Variable	*n*	OR (95% CI)	*p*	
Nasal	Age	White-Coat and Neonate	4	0.28 (0.09–0.85)	0.025	**
		Mid-Moult	6	0.62 (0.24–1.64)	0.336	
		Moulted	39	Ref		
Oral	Gender	F	15	0.46 (0.23–0.90)	0.023	**
		M	37	Ref		
	Age	White-Coat and Neonate	4	0.25 (0.08–0.74)	0.012	**
		Mid-Moult	5	0.43 (0.16–1.21)	0.109	
		Moulted	43	Ref		
Respiratory	Gender	F	35	0.59 (0.34–1.03)	0.063	*
		M	63	Ref		
	Age	White-Coat and Neonate	8	0.19 (0.08–0.44)	<0.001	**
		Mid-Moult	11	0.42 (0.18–0.95)	0.036	**
		Moulted	79	Ref		
Puncture Wounds	Age	White-Coat and Neonate	13	0.15 (0.07–0.33)	<0.001	**
		Mid-Moult	24	1.23 (0.46–3.27)	0.677	
		Moulted	104	Ref		
Abrasions	Gender	F	22	0.42 (0.23–0.76)	0.004	**
		M	53	Ref		

F = Female, M = Male, Ref = reference variable; ** = significant at *p* < 0.05, * = significant at *p* < 0.10; Age: White-Coat and Neonate were combined (Total = 39) due to low neonate count (*n* = 11).

### 3.5. Temperature

Temperature was recorded on all individuals except three (Table 3). The majority of pups were normothermic. However, almost 42% of pups displayed one form of hyperthermia, while only 5% of pups presented as hypothermic.

**Table 3:** Recorded temperatures on admissions.

Temperature Classification	Temperature Range (°C)	*n*
Marked Hypothermic	<35	5
Mildly Hypothermic	35–35.9	7
Normal	36–37.9	105
Mildly Hyperthermic	38–38.9	71
Marked Hyperthermic	>39	14
Not Recorded		3

### 3.6. Outcome

**Figure 3:**
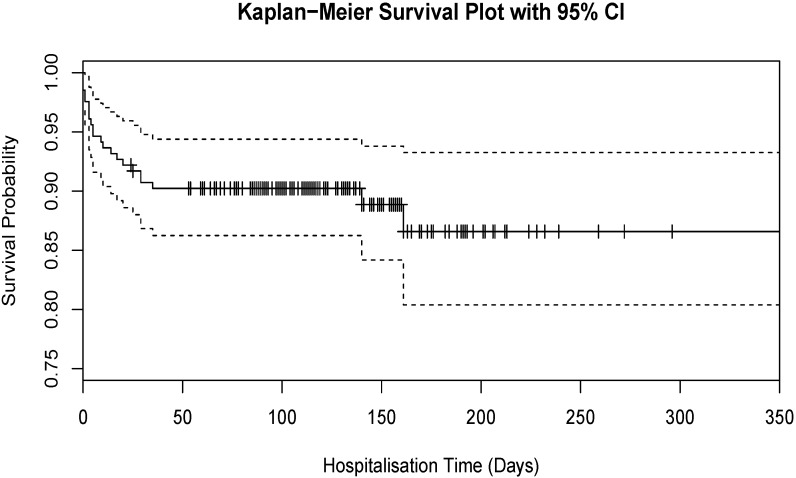
A Kaplan-Meier Survival plot of the seal rehabilitation hospitalisation time with 95% confidence intervals censored for “released”. Hatch marks indicate censorship events.

Of the 205 seals admitted for rehabilitation, 22 did not survive to release. Univariate binomial logistic regression of different variables recorded found a significant association with hospitalisation time only. The relative odds for a pup surviving until release increased by 1.06 (95% CI: 1.04–1.09, *p* < 0.001) per day during its stay in rehabilitation. A survival plot (Figure 3) demonstrates that the likelihood of a seal surviving decreased by a significant amount early in rehabilitation. There were no statistically significant associations between presenting condition and whether or not the seal was released.

### 3.7. Multivariable Binary Logistic Regression

Multivariable binary logistic regression analyses could not produce a significant model for any presenting disorder before being reduced to a univariable model. In addition, the multivariable model of outcome did not converge. The low number of seals that did not survive is most likely the cause of the non-convergence. When hospitalisation time was removed, the model did converge insignificantly. Further reductions to the model continued to produce non-significant models until all variables had been removed.

## 4. Discussion

The main purpose of this study was to examine the presenting signs of grey seal pups found during the initial admission examination. Presenting signs reported in this study, in order of prevalence, are: trauma, respiratory disorders, ocular disorders, malnourishment, oral disorders and onychia.

There was a dramatic difference in the hospitalisation time between seals that were released and those that did not survive. Examination of the survival plot shows that the majority of seals that did not survive died while in the early stages of rehabilitation. This suggests that there could be a critical period during early rehabilitation which could affect the long term outcome of the intervention. This could also indicate that current methods of long term rehabilitation are sufficient to ensure a high survivorship.

Over 80% of the seal pups had one form of trauma. The high level of trauma suggests that rehabilitation centres in the UK need to be prepared to care for wounded seals. With the recent discovery of *Mycobacterium bovis* in a non-healing wound [15] and the high prevalence of puncture wounds (68.78%), rehabilitators should be aware of the potential zoonotic risks and take appropriate protective measures when dealing with infected non-healing wounds.

Grey seal pup admission into the rehabilitation centre was seasonal. The pup production season in south-west England is mid-August through December, with the peak production occurring between August and October [1,16,17]. Admission counts peaked in November. This study found the majority of pups rehabilitated were moulted (Table 1). Pups remain on shore with their mothers until weaned at approximately 3–4 weeks of age. Rescuers are trained not to rescue a seal pup when the mother is present unless the pup has suffered serious trauma or illness [18]. After weaning and moulting, grey seal pups either enter into the water immediately or stay on land for up to a month [1]. Thus shift in the seasonality of seal admissions from pup production would not be unexpected. Non-moulted pups admitted after pup production had ended (e.g., February, March, April, May) may have been pups born post-pup production season. Alternatively, there could be delayed moulting in ill pups. Further investigation into non-moulted pups outside of pup production season is needed.

This study found that 42% of rehabilitated seals presented with hyperthermia. Grey seals spend a majority of their time in the sub-arctic waters of the north Atlantic [1]. Their adapted physiology, used to cope with the cold water, makes them highly susceptible to overheating. Thus, it is expected that a large percentage would present with hyperthermia. Rehabilitation centres should be prepared to treat for hyperthermia.

This study found a significant relationship between age and respiratory conditions. Older seal pups showed increased respiratory signs. A similar relationship with age was found with puncture wounds, oral and nasal disorders. In addition, females were less likely to have abrasions, oral, and respiratory lesions. The reasons behind these relationships are unknown, and suggest further areas of research.

Respiratory disorders increased with age. Unfortunately, this retrospective study was unable identify the cause of the respiratory disorders seen in the pups. However, Baker *et al.* [19] found an increase in pulmonary nematode parasitism in older seals, suggesting a possible cause of the increased odds of respiratory disorders in older pups. Further investigations, including tests for pulmonary nematode infestations in grey seal pups, is required.

Nasal disorders had a similar relationship to respiratory disorders. 50% of white-coat pups that presented with nasal disorders also had a respiratory disorder. This suggests that the nasal discharge observed was most likely caused by a lower respiratory tract lesion. However, this relationship was not observed with moulted pups. Naso-pharyngeal mites (*Halarachne halichoeri*) and symptomatic signs have been found in high prevalence in juvenile grey seals along the Spanish coastline [20]. Thus, it is possible that lower respiratory tract issues are more likely the cause of younger pup nasal discharge, while older pups are more likely to have an upper respiratory tract issue such as Naso-pharyngeal mites.

Puncture wounds were less likely to be found on younger pups. Puncture wounds can be caused by intraspecific interactions, predator/prey interactions, and anthropogenic causes. However, a recent study found 55% free ranging grey seal pups on a breeding beach colony had puncture wounds [21]. This suggests that intraspecific interactions are the most likely cause for puncture wounds; the seal pups had the wounds when anthropogenic and predator causes were unlikely.

Oral lesions found in this data were mostly oral ulcers. *Streptococcus equi* ssp. *zooepidemicus*, has been associated with oral ulcers in the past [19]. It has also been suggested that oral ulcers in the grey seal pup could be due to changes in the gingiva during tooth eruption [2]. Our study found older seal pups were more likely to have oral ulcers, indicating that the effect of tooth eruption could be a valid possibility. No cultures of the oral lesions were performed in this study, and thus the causative agent of the ulcers could not be determined. Further investigations need to be performed to rule out other causative agents (e.g., azotaemic, viral, or autoimmune ulcers).

It should be noted that only moulted pups had netting injuries, though the total number was low. A fishers exact showed a possible significance in this proportion, but a larger sample size is needed for rare events. Grey seal exposure to nets typically occurs in water. Only older, weaned grey seal pups enter into open water by their own choice [10]. Thus, it would be expected that only older pups would have netting injuries. Further research is needed to determine if this association holds to the expectation.

There was a large difference between the prevalence of ocular disorders in this study compared to those of Barnett *et al*. [2]. The ocular lesions noted were similar other lesions observed by Barnett *et al*. [2]. This study found 46.34% of seal pups had an ocular condition (e.g., corneal oedema, corneal ulceration, *etc.*) while in the previous study only 13% had an ocular lesion. Grey seals have large eyes, and these eyes are highly susceptible to trauma [11], which could explain the high prevalence of ocular conditions in pups presented for rehabilitation. The reason behind the difference in the studies is unknown, and should be considered by future research.

Malnourishment was found in 42.63% of seal pups, in comparison to 71% observed by Barnett *et al.* [2]. A study on grey seal mortality found that starvation was the highest probable cause of death in older juveniles [19]. The difference is likely due to different methods used to define malnourishment. This retrospective study relied on the subjective BCS, while Barnett *et al.* [2] used weights and muscle mass cover and Baker *et al.* [19] used post mortem information. The BCS was not standardised before the study, and thus may have caused an under-reporting of malnourished prevalence. Alternatively, the use of weight in sick animals to assess nutritional status may have caused an over-estimation of the malnourished prevalence in Barnett *et al.* [2]. In addition, weight of the pup might reflect the age of the mother instead of the nutritional status of the pup [22]. Further research is needed to determine the true malnourishment prevalence.

There were some limitations to this study due to its retrospective nature. The examination of the seals was not standardised and the examiner on admission was not noted in the record, thus inter-observer reliability could not be assessed. In addition, this study performed multiple comparisons and increased the familywise error rate. However, due to the nature of this exploratory epidemiological survey and the data, no multiplicity adjustment is required [23,24]. Confirmatory studies for this study’s findings with appropriate controls should be performed in the future.

## 5. Conclusions

The rehabilitation of grey seals will continue in the UK in the foreseeable future. The stranding network for seals must maintain a well-equipped and informed system. With climate change, diseases in wild populations are expected to change [5,6,7]. This study presents an updated prevalence of presenting conditions of seal pups admitted to rehabilitation. Continued assessment of disease prevalence and presenting conditions on admission will allow rescuers to prepare for the continued rehabilitation of grey seals.

## Supplementary Materials

Supplementary File 1
