# Supplementary material for: Prevalence of Presenting Conditions in Grey Seal Pups (Halichoerus grypus) Admitted for Rehabilitation
Source: Vet Sci. 2015 Jan 5;2(1):1–11. doi: 10.3390/vetsci2010001 (PMC5644611; doi:10.3390/vetsci2010001)
Supplement: Supplementary File 1 [file vetsci-02-00001-s001.docx]

Supplementary Materials

**Table S1.** Insignificant Univariable Binary Logistic Regressions of each presenting disorder observed in rehabilitated grey seal pups.

| **Disorder** | **Factor** | **Variable** | ***n*** | **OR (95% CI)** | ***p*** |
| --- | --- | --- | --- | --- | --- |
| Ocular | Gender | F | 45 | 1.46 (0.83–2.54) | 0.185 |
|  |  | M | 50 | Ref |  |
|  | Age | White-Coat and Neonates | 17 | 0.77 (0.38–1.85) | 0.481 |
|  |  | Mid-Moult | 10 | 0.50 (0.22–1.15) | 0.102 |
|  |  | Moulted | 68 | Ref |  |
| Nasal | Gender | F | 19 | 0.82 (0.43–1.58) | 0.552 |
|  |  | M | 30 | Ref |  |
| Abrasions | Age | White-Coat and Neonates | 15 | 1.18 (0.57–2.47) | 0.653 |
|  |  | Mid-Moult | 13 | 1.45 (0.65–3.24) | 0.367 |
|  |  | Moulted | 47 | Ref |  |
| Orthopaedic | Gender | F | 10 | 0.96 (0.41–2.29) | 0.935 |
|  |  | M | 14 | Ref |  |
|  | Age ^†^ | Not Moulted | 5 | 0.48 (0.17–1.35) | 0.164 |
|  |  | Moulted | 19 | Ref |  |
| Netting Injuries | Gender | F | 5 | Ref |  |
|  |  | M | 2 | 0.28 (0.05–1.49) | 0.137 |
|  | Age | White-Coat and Neonates | 0 | ^‡^ |  |
|  |  | Mid-Moult | 0 | ^‡^ |  |
|  |  | Moulted | 7 |  |  |
| Onychia | Gender | F | 8 | 0.99 (0.38–2.56) | 0.975 |
|  |  | M | 11 | Ref |  |
|  | Age | White-Coat and Neonates | 3 | 0.79 (0.21–2.92) | 0.722 |
|  |  | Mid-Moult | 3 | 1.05 (0.28–3.95) | 0.941 |
|  |  | Moulted | 13 | Ref |  |
| Malnourishment | Gender | F | 34 | 1.07 (0.60–1.92) | 0.824 |
|  |  | M | 47 | Ref |  |
|  | Age | White-Coat and Neonates | 13 | 0.81 (0.37–1.78) | 0.603 |
|  |  | Mid-Moult | 9 | 0.53 (0.23–1.26) | 0.153 |
|  |  | Moulted | 59 | Ref |  |

F = Female, M = Male, Ref = reference variable; White-Coat and Neonates combined due to low number of neonates (*n* = 11); ^†^ Age was combine into two classes due to low number of white-coats and neonates (*n* = 0);
^‡^ Unable to perform model due to no pups in all age groups except moulted.

**Table S2.** Univariable Binary Logistic Regressions of rehabilitated grey seal pups surviving to release.

| **Variable** | **Group** | ***n*** | **Total** | **%** | | **Mean ± S.D.** | **OR (95% CI)** | ***p*** |
| --- | --- | --- | --- | --- | --- | --- | --- | --- |
| Hospitalisation Time |  |  |  | |  | 128.58 ± 50.8 | 1.06 (1.04–1.09) | <0.001 |
| Transit Time |  |  |  | |  | 0.82 ± 0.5 | 0.90 (0.35–2.29) | 0.826 |
| Ocular | Absent | 99 | 110 | | 90.00 |  | Ref |  |
|  | Present | 84 | 95 | | 88.42 |  | 0.85 (0.35–2.06) | 0.716 |
| Nasal | Absent | 140 | 156 | | 89.74 |  | Ref |  |
|  | Present | 43 | 49 | | 87.76 |  | 0.82 (0.30–2.22) | 0.695 |
| Oral | Absent | 139 | 153 | | 90.85 |  | Ref |  |
|  | Present | 44 | 52 | | 84.62 |  | 0.55 (0.22–1.41) | 0.214 |
| Respiratory | Absent | 97 | 107 | | 90.65 |  | Ref |  |
|  | Present | 86 | 98 | | 87.76 |  | 0.74 (0.30–1.80) | 0.504 |
| Puncture Wounds | Absent | 57 | 64 | | 89.06 |  | 0.97 ( 0.39–2.66) | 0.949 |
|  | Present | 126 | 141 | | 89.36 |  | Ref |  |
| Abrasions | Absent | 116 | 130 | | 89.23 |  | Ref |  |
|  | Present | 67 | 75 | | 89.33 |  | 1.01 (0.40–2.53) | 0.982 |
| Skeletal | Absent | 162 | 181 | | 89.50 |  | Ref |  |
|  | Present | 21 | 24 | | 87.50 |  | 0.82 (0.22–3.01) | 0.766 |
| Netting Injury | Absent | 176 | 198 | | 88.89 |  |  |  |
|  | Present | 7 | 7 | | 100 |  | ^‡^ |  |
| Onychia | Absent | 166 | 186 | | 89.25 |  | Ref |  |
|  | Present | 17 | 19 | | 89.47 |  | 1.02 (0.22–4.76) | 0.976 |
| Malnourishment | Absent | 98 | 109 | | 89.91 |  | Ref |  |
|  | Present | 70 | 81 | | 86.42 |  | 0.71 (0.29–1.74) | 0.459 |
| Age | White-coat | 33 | 39 | | 84.62 |  | 0.53 (0.19–1.52) | 0.240 |
|  | Mid-moult | 26 | 30 | | 86.67 |  | 0.63 (0.19–2.11) | 0.452 |
|  | Moulted | 124 | 136 | | 91.18 |  | Ref |  |
| Gender | F | 81 | 87 | | 93.10 |  | 2.12 (0.79–5.66) | 0.134 |
|  | M | 102 | 118 | | 86.44 |  | Ref |  |

OR = Odds Ratio, CI = Confidence Interval, S.D. = Standard Deviation; ^‡^ model not able to be performed due to 0% not surviving.
